# Dysregulated transcriptional and post-translational control of DNA methyltransferases in cancer

**DOI:** 10.1186/2045-3701-4-46

**Published:** 2014-08-19

**Authors:** Ruo-Kai Lin, Yi-Ching Wang

**Affiliations:** 1 Graduate Institute of Pharmacognosy, Taipei Medical University, 250 Wu-Hsing Street, Taipei 110, Taiwan; 2 Department of Pharmacology and Institute of Basic Medical Sciences, National Cheng Kung University, No.1, University Road, Tainan 70101, Taiwan; 3 Program for the Clinical Drug Discovery from Botanical Herbs, Taipei Medical University, 250 Wu-Hsing Street, Taipei 110, Taiwan; 4 Program for Clinical Pharmacogenomics and Pharmacoproteomics, Taipei Medical University, 250 Wu-Hsing Street, Taipei 110, Taiwan

**Keywords:** DNA methyltransferase, Cancer, Tumor suppressor gene, Transcription, Post-translational modifications

## Abstract

Cancer is a leading cause of death worldwide. Aberrant promoter hypermethylation of CpG islands associated with tumor suppressor genes can lead to transcriptional silencing and result in tumorigenesis. DNA methyltransferases (DNMTs) are the enzymes responsible for DNA methylation and have been reported to be over-expressed in various cancers. This review highlights the current status of transcriptional and post-translational regulation of the DNMT expression and activity with a focus on dysregulation involved in tumorigenesis. The transcriptional up-regulation of *DNMT* gene expression can be induced by Ras-c-Jun signaling pathway, Sp1 and Sp3 zinc finger proteins and virus oncoproteins. Transcriptional repression on *DNMT* genes has also been reported for p53, RB and FOXO3a transcriptional regulators and corepressors. In addition, the low expressions of microRNAs 29 family, 143, 148a and 152 are associated with DNMTs overexpression in various cancers. Several important post-translational modifications including acetylation and phosphorylation have been reported to mediate protein stability and activity of the DNMTs especially DNMT1. In this review, we also discuss drugs targeting DNMT protein expression and activation for therapeutic strategy against cancer.

## Introduction

Cancer is a leading cause of death worldwide, accounting for 8.2 million deaths in 2012 [1]. The process of tumorigenesis needs to be initiated and promoted by molecular abnormalities including oncogenes activation and tumor suppressor genes (TSGs) inactivation [2]. Methylation of CpG islands is one of the epigenetic modifications in mammalian genome that modulates gene expression without changes in the DNA sequence [2]. Aberrant promoter hypermethylation of CpG islands associated with TSGs can lead to transcriptional silencing and result in tumorigenesis. DNA methylation is frequently not restricted to a single CpG island but affects multiple independent loci, reflective of a widespread deregulation of DNA methylation pattern in different types of tumors [3,4]. Development of genome-wide high-throughput technologies has facilitated the identification of global DNA methylation pattern [5,6]. For example, genomic screening of 98 different primary human tumors has revealed that on an average there exist about 600 aberrantly methylated CpG islands in each tumor [7]. In addition, an increase of methylation variability may contribute to tumor heterogeneity [8]. Collectively, dysregulation of DNA methylation is apparently one of the major barriers to effective cancer diagnosis and treatment in different types of cancer.

Epigenetic disorders give rise to several significant human diseases including various cancers, neuron disorder, psychosis, and cardiovascular diseases, many of which are associated with altered expression and activity of DNA methyltransferases (DNMTs) [9-13]. DNMTs are the enzymes responsible for DNA methylation through transfer of methyl group to cytosine residue of CpGs [2]. Five types of DNMTs have been identified, viz. DNMT1, 2, 3A, 3B, and 3L. DNMT1 comprises a large N-terminal domain with regulatory function and a smaller C-terminal catalytic domain [14]. The regulatory domain harbors different motifs and is involved in the intracellular delivery and regulation of catalytic activity of DNMT1. DNMT1 has been shown to prefer hemimethylated over unmethylated DNA 30- to 40-fold *in vitro*[15-17]. It is referred to as a “maintenance” methyltransferase and is the primary enzyme responsible for copying the methylation patterns after DNA replication. DNMT1 localizes to replication foci and interacts with PCNA, a processivity factor for DNA replication complex [18]. However, evidences show that DNMT1 may also work together with DNMT3A and DNMT3B in *de novo* methyltransferase activity in certain genome in both embryonic cells and differentiated somatic cells [19,20]. Many interacting proteins have been reported to bind to their N-terminal region by biochemical interaction assay [14]. For example, DNMT1 directly interacts with histone modifying enzymes such as histone H3K9 methyltransferase SUV39H1, histone H3K27 methyltransferase EZH2, and histone deacytelase HDAC1 and HDAC2 [14,21]. DNMT1 also interacts with methyl-CpG-binding proteins such as MBD2, MBD3 and MeCP2 and with the heterochromatin binding protein HP1 [14].

Notably, DNMT1, DNMT3A, and DNMT3B are overexpressed in a coordinate manner in most tumor tissues and at a significantly higher level in cancer than in non-tumorous tissues [22-24]. The mechanism underlying DNMTs overexpression is worthy of comprehensive discussion. Delineating mechanisms of DNMTs overexpression will provide more information and strategies to remedy the altered epigenetic states. It will offer more exciting opportunities that can reactivate epigenetically silenced TSGs and critical anti-cancer pathways [25].

## Transcriptional regulation of *DNMT* gene expression

**Figure 1:**
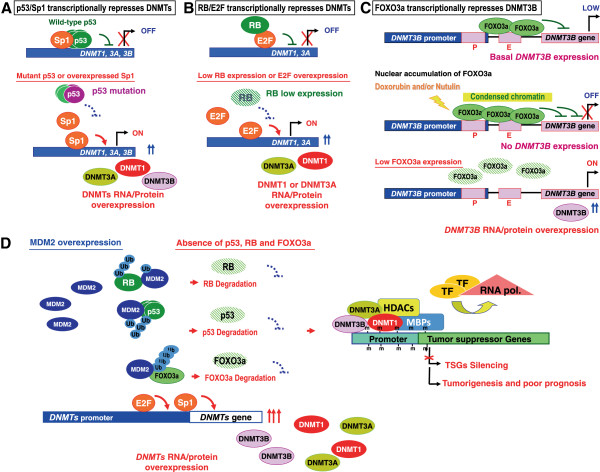
**Transcriptional regulation on *DNMT *gene expression. (A)** p53 transcriptionally suppresses *DNMTs* through binding with Sp1 protein to the *DNMT1, 3A* and *3B* promoters. **(B)** RB transcriptionally suppresses *DNMT1/3A* through binding with E2F1 protein to the *DNMT1* and *3A* promoters. **(C)** FOXO3a binds to the *FOXO3a* DNA element of the *DNMT3B* promoter to repress *DNMT3B* transcription. **(D)** Clinically, overexpressed MDM2 dramatically induces *DNMT1, DNMT3A,* and *DNMT3B* expression by negative control over p53, RB and FOXO3a leading to methylation of multiple TSGs and tumorigenesis.

The earlier study on transcriptional regulation of *DNMT* mediated by Ras-c-Jun signaling pathway provided a molecular explanation for the role of DNMT1 to carcinogenesis [26,27]. The expressions of *DNMT1*, *DNMT3A* and *DNMT3B* genes are also controlled by Sp1 and Sp3 zinc finger proteins [28,29]. Wilms' tumour 1 protein has been shown to directly transactivate *DNMT3A* expression [30]. Homeobox B3 can bind to and activate *DNMT3B* gene [31]. In addition to transcription factors, several important transcriptional repressors have been reported to suppress the *DNMT1*, *DNMT3A* and *DNMT3B* gene expression, including p53, RB and FOXO3a (Table 1 and Figure 1). The major findings are described below.

**Table 1:** **Transcriptional regulation of *DNMT *** **promoter activity and/or mRNA expression**

**Pathways**	**Mechanisms**	***DNMTs *** **mRNA/promoters**	**References**
**Down regulation**			
p53	p53/Sp1 transcriptional repression	DNMT1/3A/3B	[32]
RB/E2F	RB/E2F transcriptional repression	DNMT1/3A	[33-36]
FOXO3a	Transcriptional repression	DNMT3B	[37]
**Up regulation**			
Ras/AP-1	AP1 transcriptional activation	DNMT1	[26,27,38]
Sp1	Transcriptional activation	DNMT1/3A/3B	[28,29,32]
Sp3	Transcriptional activation	DNMT1/3A/3B	[28,29]
E2F	Transcriptional activation	DNMT1	[34,39]
ERK	Unknow	DNMT1/3A	[40]
17*β*-estradiol	ER-dependent transcription activation	DNMT3B	[41]
Homeobox B3	Promoter binding	DNMT3B	[31]
Wilms' tumour 1	Transcriptional activation	DNMT3A	[30]
**Viruse induction**			
LMP1	Activation of JNK/AP-1 pathway	DNMT1	[42]
BKV Tag and E1a	pRB/E2F pathway	DNMT1	[43]
HBx	Promoter transcriptional activator	DNMT1/3A	[44]
HBx	Promoter transcriptional repression	DNMT3b	[44]
HIV-1	Through transcription factor AP-1	DNMT1	[45]

LMP1: latent membrane protein 1.

BKV Tag and E1a: Human polyomavirus BKV large T antigen and adenovirus E1a.

HBx: Hepatitis B virus X protein.

HIV-1: early expressed HIV-1 proteins.

### The p53-mediated regulation of *DNMT* genes

The tumor suppressor gene *p53* encodes a transcription factor that mediates many downstream effects such as growth arrest and apoptosis through activation or repression of its target genes [46]. However, the *p53* gene is a frequent target of missense mutation rendering it unable to recognize the p53 consensus binding sites therefore loss of transcriptional function of p53 in cancers [47]. Sequencing analyses reveal that point mutation and small intragenic deletion/insertion of *p53* gene are significantly associated with DNMT1 protein overexpression [32]. A study shows that deletion of *p53* in the HCT116 human colon carcinoma cell line results in increase of DNMT1 mRNA and protein [48]. Wild-type p53 decreases *DNMT1* promoter activity and expression level through the exon 1 region (-19 to +317) of *DNMT1* promoter, which contains p53 putative binding sites, in lung cancer cells [32,48]. In addition, wild-type p53 protein has been shown to negatively regulate DNMT1 expression by forming a complex with Sp1 protein and chromatin modifiers on the *DNMT1* promoter [32]. Low level of exogenous Sp1 expression enhances the repressive activity of endogenous p53 on the *DNMT1* promoter, whereas a high level of Sp1 expression upregulates *DNMT1* gene expression level in A549 (p53 wild-type) cells. In H1299 (p53 null) cells, exogenous Sp1 induces DNMT1 expression in a dose-dependent manner. A high level of Sp1, via its COOH-terminal domain, induces interaction between p53 and MDM2, resulting in degradation of p53 by MDM2-mediated ubiquitination [32]. Clinical data from 102 lung cancer patients indicated that overexpression of DNMT1 is significantly associated with *p53* mutation and high expression of Sp1 protein. In addition, patients with overexpression of both DNMT1 and Sp1 proteins show poor prognosis [32]. Cell and clinical data provide evidence that deregulation of DNMT1 is associated with gain of transcriptional activation of Sp1 and/or loss of repression of p53. DNMT1 overexpression is involved in epigenetic alterations of multiple TSGs that ultimately leads to lung tumorigenesis and poor prognosis [32].

Some reports have also shown that Sp1 and Sp3 increase the activity of *DNMT1, DNMT3A,* and *DNMT3B* promoters by physical binding to their promoters in mouse NIH3T3 cells or human embryonic kidney 293T cells [28,29]. p53 is shown to suppress the promoter activity and mRNA/protein expression of *DNMT3A* through binding to its promoter and the suppression can be attenuated by knockdown of p53 [33]. Whether overexpression of DNMT3A and DNMT3B resulted from the loss of transcriptional regulation of p53/Sp1 warrants further investigation. It is justifiable to propose that overexpression of DNMTs is associated with the gain of transcriptional activation of Sp1 and/or the loss of repression of p53 (Figure 1A).

### The RB-mediated regulation of *DNMT* genes

The RB (retinoblastoma) protein is a tumor suppressor, which plays a pivotal role in the negative control of the cell cycle and in tumor progression [49]. The RB protein represses gene transcription, required for transition from G1 to S phase, by directly binding to the transactivation domain of E2F and by binding to the promoter of the target genes as a complex with E2F [50]. RB also represses transcription by remodeling chromatin structure through interaction with proteins such as HP1, SWI/SNF, HDAC1 and SUV39H1, which are involved in DNA methylation, nucleosome remodeling, histone deacetylation and histone methylation, respectively [51-54].

The mouse and human *DNMT1* promoters are found to contain E2F binding sites that are required for RB/E2F regulation in wtPrE (wild-type prostate epithelial cell line) [34]. *DNMT1* is negatively regulated by E2F-RB-HDAC pathway in mouse NIH3T3 embryonic fibroblast, monkey COS-7 kidney cell, and saos-2 human osteosarcoma cell lines [35]. In addition, *DNMT1* mRNA can be diminished by overexpression of RB protein in saos-2 cells and are induced by deletion of *RB* gene in wtPrE cells [34,35]. RB also suppresses *DNMT3A* promoter activity and mRNA/protein expression through binding with E2F1 protein to the *DNMT3A* promoter [36]. Repression of DNMT3A by RB leads to the decrease of methylation level globally and TSG specifically, such as *RARβ*, *FHIT*, and *RASSF1A* genes [36]. Together, these data suggest that RB is a transcriptional repressor of *DNMT1* and *DNMT3A* genes (Figure 1B).

### The FOXO3a-mediated regulation of *DNMT* genes

Forkhead O transcription factor 3a (FOXO3a) belongs to a large protein family of transcriptional regulators characterized by a conserved DNA-binding domain termed the “forkhead-box” [55]. To date, many reports indicate a tumor suppressor role for FOXO3a. For example, ectopic overexpression of FOXO3a significantly impairs tumor growth in cell and xenograft models in breast cancer and promotes apoptosis in leukemia and prostate cancer cells [56,57]. In addition, restrained transcriptional activity of FOXO3a in cancer cells results in promoting angiogenesis and tumor progression [58-60]. FOXO3a has been shown to transcriptionally up-regulate apoptotic-related gene such as *p27kip*[61], *Bim*[62], and *Fas* ligand [63]. In contrast, FOXO3a could transcriptionally repress microRNA21, which suppresses the expression of *Fas* ligand [64]. Of note, the gene deletion of *FOXO3a* is found in early-stage lung adenocarcinoma in smokers and tobacco carcinogen-initiated lung tumors in mice [37,65]. Restoration of FOXO3a in FOXO3a-deficient lung cancer cells increases the cell apoptosis response to nicotine-derived nitrosamino ketone-mediated DNA damage [66]. The last-mentioned two studies implicate that loss of FOXO3a may contribute to lung cancer pathogenesis.

We recently showed that FOXO3a negatively regulates *DNMT3B* promoter activity by interacting with the binding element *FOXO3a* (+166 ~ +173) of *DNMT3B* promoter [67]. Ectopically overexpressed FOXO3a or combined treatment with doxorubicin to induce FOXO3a nuclear accumulation leads to further binding at the distal *FOXO3a* site (-249 ~ -242). Abundant FOXO3a represses *DNMT3B* promoter by establishing a repressed chromatin structure, while knockdown of FOXO3a results in an open chromatin structure and high DNMT3B mRNA and protein expression. Importantly, enforced abundant nuclear accumulation of FOXO3a could decrease expression of DNMT3B with synergistic inhibition of tumor growth and decrease in methylation status on TSGs in human lung tumor xenograft specimens [67]. It is plausible that FOXO3a binds to the *FOXO3a* DNA element of the *DNMT3B* promoter to repress *DNMT3B* expression (Figure 1C).

### Transcriptional deregulation of *DNMT* genes by MDM2 overexpression

p53 protein is known to be degraded in cytoplasm by ubiquitin-mediated proteasomal degradation pathway modulated by MDM2 [68]. MDM2, an E3 ubiquitin ligase, also physically interacts with RB and FOXO3a resulting in degradation of RB and FOXO3a proteins [69,70]. Overexpression of MDM2 has been demonstrated in many human cancers [36,71]. In addition, oncogenic ERK phosphorylates FOXO3a at Ser^294^, Ser^344^, and Ser^425^ thereby enhancing the interaction with MDM2 and results in promoting degradation of FOXO3a [69]. Therefore, we hypothesized that MDM2 plays a critical role in regulating the *DNMT* genes by synergistically destabilizing p53, RB and FOXO3a proteins. To test this hypothesis we analyzed the relationship of MDM2 protein with p53, RB, FOXO3a and DNMT proteins in lung cancer cell, xenograft and patient models. Dramatic induction of *DNMT3A* and *DNMT3B* expression by ectopic overexpression MDM2 suggests a negative control of MDM2 over RB and FOXO3a [36,67]. Note that treatment with the MDM2 inhibitor, Nutlin-3, significantly reduces DNMT3A and DNMT3B expression and methylation of TSGs, as well as tumor growth *in vivo*[36,67]. Clinically, MDM2 overexpression inversely correlates with expression of p53, RB and FOXO3a proteins in tumor tissues from lung cancer patients. Importantly, a sub-group of patient with gene expression signature of DNMTs high, p53/RB/FOXO3a low, and MDM2 high expression profile correlating with poor survival [33,36,67]. This defined signature may serve as a prognostic marker in lung cancer patients whose genomic DNA may exert promoter hypermethylation in multiple TSGs (Figure 1D).

## The microRNA-mediated regulation of DNMTs

MicroRNAs (miRs) are small, noncoding RNAs that regulate expression of many genes. Recent studies suggest that abnormal expressions of miRs are involved in pathogenesis of different types of human cancers [72]. Previous reports have shown that expression profiles of miRs in lung cancer are different from normal lung. The miR-29 family (29a, 29b, and 29c) has intriguing complementarities to the 3'-UTRs of *DNM3A* and *DNMT3B*[73]. The expression of miR-29s is inversely correlated to *DNMT3A* and *DNMT3B* in lung cancer tissues, and miR-29s directly target the 3'-UTRs of both *DNMT3A* and *DNMT3B*. The enforced expression of miR-29s in lung cancer cell lines restores normal patterns of DNA methylation. The miR-29s further induces re-expression of methylation-silenced TSGs, such as *FHIT* and *WWOX*, and inhibits tumorigenicity *in vitro* and *in vivo*[73]. Enforced miR-29b expression in acute myeloid leukemia cells also results in marked reduction in the expression of DNMT1, DNMT3A, and DNMT3B and ultimately to re-expression of *p15*^*INK4b*^ and *ESR1* via promoter DNA hypomethylation [74]. Of note, an inverse correlation between miR-29c expression and DNMT3A and DNMT3B protein expression has been reported in melanomas [75].

In addition to miR-29s, ectopic expression of miRNA-148a in lung cancer cell lines also results in a significant reduction in the expression of DNMT1 [76]. Using luciferase reporter assay, *DNMT1* mRNA was found to be a target of miR-148b and miR-152 [77]. Antagomir-mediated knock-down and re-expression of miRs assays support that miR-148b, miR-29c, and miR-26b down-regulate *DNMT3B* gene in breast cancer cells [78]. Furthermore, overexpression of miR-148b and -152 in pancreatic cancer cell lines decreases DNMT1 expression, restores normal DNA methylation patterns and induces re-expression of TSGs, like *BNIP3 *and *SPARC*[77]. It is to be noted that miR-143 was reported to directly target DNMT3A. In colorectal cancer tissues, the miR-143 expression was observed to be inversely correlated with DNMT3A mRNA and protein expression [79]. Specifically, miR-1741, miR-16c, miR-222 and miR-1632 are found to influence expression of *DNMT3A* or *DNMT3B*, possibly through their 3′-UTR post-transcriptional regulation [80]. Table 2 summarizes the regulation of DNMTs by miRs.

**Table 2:** Regulation of DNMT expression by miRNAs

**Pathway**	**Regulated regions**	**DNMTs**	**Cancer types**	**References**
miR-16c	3'-UTRs	DNMT3B	*in vitro*	[80]
miR-26b	ND	DNMT3B	breast cancer	[78]
miR-29a	3'-UTRs	DNMT3A/3B	lung cancer	[73]
miR-29b	3'-UTRs	DNMT1/3A/3B	lung, ALL and melanomas	[73,74]
miR-29c	3'-UTRs	DNMT3A/3B	breast, lung and melanomas	[73,75,78]
miR-143	3'-UTRs	DNMT3A	colorectal	[79]
miR-148a	ND	DNMT1	lung and pancreas	[76,77]
miR-148b	ND	DNMT3B	breast cancer	[78]
miR-152	ND	DNMT1	pancreas	[77]
miR-222	3'-UTRs	DNMT3B	*in vitro*	[80]
miR-1632	3'-UTRs	DNMT3B	*in vitro*	[80]
miR-1741	3'-UTRs	DNMT3A	*in vitro*	[80]

ND: non-determined.

ALL: Acute lymphoblastic leukemia.

## Post-translational modification of DNMT proteins

**Figure 2:**
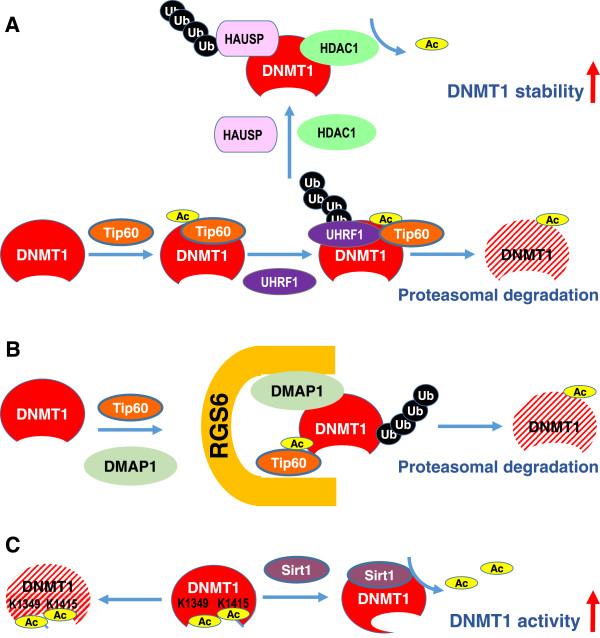
**Acetylation-mediated DNMT stability and activity. (A)** Tip60 promotes acetylation of DNMT1, which triggers ubiquitination by the E3 ligase UHRF1, thereby targeting DNMT1 for proteasomal degradation. **(B)** RGS6 serves as a scaffold to facilitate Tip60 acetylation of DNMT1 and subsequent DNMT1 degradation. **(C)** Deacetylation of Lys1349 and Lys1415 by SIRT1 in the catalytic domain of DNMT1 enhances the methyltransferase activity of DNMT1.

**Figure 3:**
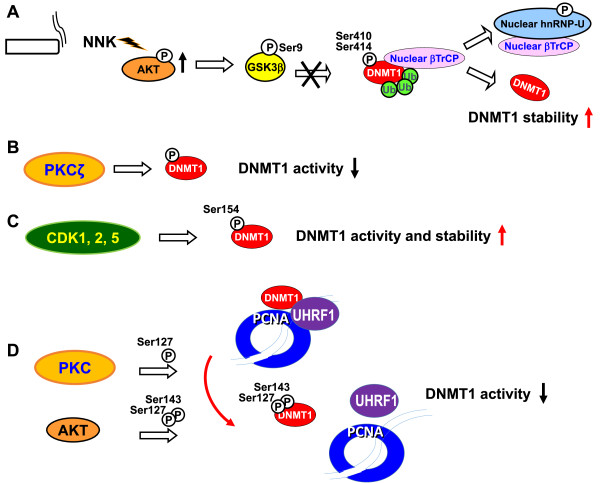
**Phosphorylation-mediated DNMT stability and activity. (A)** Cigarette carcinogen NNK activates AKT, inhibits GSK3β-mediated DNMT1 phosphorylation at Ser410 and Ser414, thereby leading to DNMT1 protein accumulation. **(B)** Phosphorylation of DNMT1 by PKCζ reduces its methyltransferase activity. **(C)** Phosphorylation of DNMT1 at Ser154 by CDK1, 2 and 5 enhances enzymatic activity and protein stability of DNMT1. **(D)** Phosphorylation of DNMT1 at Ser127 and/or Ser143 decreases its interaction with UHRF1 ubiquitin-like protein and renders DNMT1 less efficient to catalyze the DNA methyltransferase activity at the replication fork.

Several important post-translational modification including acetylation and phosphorylation have been reported to mediate protein stability and activity of the DNMTs especially DNMT1 (Figures 2 and 3). The major findings are described below.

### Acetylation-mediated DNMT stability and activity

DNMT1 has been shown to be destabilized by acetylation-mediated ubiquitination. DNMT1 physically interacts with acetyltransferase Tip60, herpesvirus-associated ubiquitin specific protease (HAUSP), ubiquitin-like containing PHD and RING finger domains 1 (UHRF1), HDAC1 and PCNA on chromatin [81]. Tip60 promotes acetylation of DNMT1, which triggers ubiquitination by the E3 ligase UHRF1, thereby targeting DNMT1 for proteasomal degradation during late S phase [82]. Conversely, HAUSP and HDAC1 protect DNMT1 from degradation through deubiquitination and deacetylation, respectively [81] (Figure 2A). In addition, the pleiotropic regulator of G protein signaling (RGS) family member RGS6 facilitates Tip60-mediated degradation of DNMT1 [83]. RGS6 may serve as a scaffold of Tip60, DNMT1 and Dnmt1-associated protein (DMAP1) to facilitate Tip60 acetylation of DNMT1 and subsequent DNMT1 polyubiquitylation and degradation [83] (Figure 2B). In contrast, the histone deacetylase SIRT1 physically associates with DNMT1 and deacetylates acetylated DNMT1 *in vitr*o and *in vivo*[84]. Using mass spectrometry analysis, 12 new acetylated lysine sites are identified in DNMT1 [85]. Deacetylation of different lysines on DNMT1 by SIRT1 has different effects on the functions of DNMT1. For example, deacetylation of Lys1349 and Lys1415 by SIRT1 in the catalytic domain of DNMT1 enhances the methyltransferase activity of DNMT1 (Figure 2C). Collectively, these findings suggest that deacetylation of the identified acetylated lysine sites in DNMT1 may be involved in the impaired activity of DNMT1.

### Phosphorylation-mediated DNMT stability and activity

Lin *et al.* found that the tobacco-specific nitrosamine 4-(methylnitrosamino)-1-(3-pyridyl)-1-butanone (NNK) increases DNMT1 protein expression and activity [86]. Strong nuclear staining of DNMT1 protein in lung tumor tissues is significantly associated with smoking status of lung cancer patients [22,86]. Cigarette smoking is a dominant risk factor for lung cancer. Among the multiple components of tobacco smoke, 20 carcinogens convincingly cause lung tumors in laboratory animals or humans. Of these, NNK is likely to play a major role because NNK has been shown to induce lung tumor in rats, mice and hamsters [87]. In addition, exposure of NNK not only causes gene mutation, but also stimulates the promoter hypermethylation on multiple TSGs in bladder, liver, and lung cancers, including *FHIT*, *RASSF1A*, and *p16*^*INK4A*^*, DAPK1*, *RUNX3*, *RARβ* and *SFRP* genes [88-93]. Notably, NNK stimulates the AKT, NF-κB, EGFR, and ERK1/2 signal pathways resulting in increased cell proliferation and survival [94-96]. It has previously been shown that AKT inactivates GSK3β Ser/Thr kinase, which phosphorylates its substrate protein and recruits the E3-ubiqutin ligase βTrCP, leading to substrate degradation [97,98]. In addition, stabilization of DNMT1 protein is regulated by inhibiting GSK3β-mediated phosphorylation and proteasomal degradation of DNMT1 upon activation of T cell receptor signaling [99]. Lin *et al.* showed that GSK3β interacts with DNMT1 to phosphorylate DNMT1 at Ser410 and Ser414 and promotes binding of DNMT1 by βTrCP leading to proteasomal degradation of DNMT1. They also demonstrated that DNMT1 contains a domain, ESGXXS, similar to the conserved binding motif DSGXXS of βTrCP [86]. This study suggests that NNK activates AKT, then inhibits GSK3β/βTrCP–mediated protein degradation, leading to DNMT1 protein accumulation [86]. In addition, DNMT1/βTrCP interaction could be interrupted by treating cells with NNK. NNK also induces βTrCP translocation to the cytoplasm by interacting with phosphorylated heterogeneous nuclear ribonucleoprotein U (hnRNP-U) shuttling protein mediated by AKT. Therefore, NNK exposure results in DNMT1 nuclear accumulation and hypermethylation of the promoters of multiple TSGs [86]. Figure 3A shows the phosphorylation-mediated DNMT stability control induced by the cigarette carcinogen NNK.

In addition to Ser410 and Ser414 of DNMT1, recent studies have suggested that the enzymatic activity of DNMT1 is possibly modulated by phosphorylation of Ser/Thr residues located in the N-terminal domain of the enzyme [100-102]. Protein kinase C (PKC) α, βI, βII, δ, γ, η, ζ and μ preferentially phosphorylate the N-terminal domain of human DNMT1 [102]. Phosphorylation of DNMT1 by PKCζ reduces its methyltransferase activity *in vitro*[102] (Figure 3B). In addition, phosphorylation of DNMT1 at Ser154 by CDKs, including CDK1, 2 and 5, is important to enhance enzymatic activity and protein stability of DNMT1 [100] (Figure 3C). AKT and PKC are capable of phosphorylating DNMT1 at the residues Ser127/143 and Ser127, respectively [101]. Phosphorylation of the DNMT1 at Ser127 and/or Ser143 decreases the capacity of the protein to interact with PCNA and UHRF1 proteins and renders DNMT1 less efficient to catalyze methylation [101] (Figure 3D). Interestingly, phosphorylation of DNMT1 at Ser143 by AKT1 interferes with the methylation of Lys142 by SET7, a known histone methyltransferase involved in proteasome-mediated degradation of DNMT1 [103].

## The impact of viruses on the regulation of *DNMT* genes

Several viruses have been reported to increase DNMTs expression (Table 1). Epstein-Barr virus (EBV) is closely associated with human malignancies, including nasopharyngeal carcinoma, Burkitt's lymphoma, T-cell lymphoma, gastric carcinoma [104,105]. Epigenetic regulation of EBV plays a central role in viral latency and viral-associated carcinogenesis [105]. EBV latent membrane protein 1 (LMP1) activates cellular DNMTs, resulting in hypermethylation and silencing of *E-cadherin*. LMP1-mediated DNMT1 activation involves JNK but not NF-κB and p38 mitogen-activated protein kinases [42]. The EBV oncogene product LMP1, induces promoter hypermethylation of *RARβ2* via up-regulation of DNMT1, DNMT3A, and DNMT3B proteins, leading to decrease in RARβ2 expression in nasopharyngeal carcinoma cell lines [43]. Human polyomavirus BKV large T antigen and adenovirus E1a also strongly increase DNMT1 expression. Mutation of the E2F sites within the *DNMT1* promoter dramatically abrogates transcriptional activation, suggesting that BKV viral induction of DNMT1 may be through modulation of pRB/E2F pathway [39].

The hepatitis B virus (HBV) X protein (HBx) plays a key role in the molecular pathogenesis of HBV-related hepatocellular carcinoma. HBx expression increases total DNMT activities and selectively promotes regional hypermethylation of specific TSGs, including *RASSF1A*, *GSTP1*, and *CDKN2B*, in pHBx-transfected cells [44]. Another study shows that enforced HBx suppresses *RASSF1A* possibly via induction of DNMT1 and DNMT3B expression [106].

Human immunodeficiency virus type 1 (HIV-1) also has been reported to induce DNMT1 through the responsive element residing in the -1634 to +71 of *DNMT1* promoter [45]. The increase in expression of DNMT1 and overall genomic methylation as well as hypermethylation of the *p16*^*INK4A*^ gene are found when infected with HIV-1 in Hut 78 lymphoid cells [107]. HIV infection of human regulatory T cells down-regulates FOXP3 expression mediated by increasing DNMT3B levels and DNA methylation in the *FOXP3* gene [108]. Therefore, the ability of increased DNMT activity to downregulate the expression of critical genes may be one of the mechanisms for dysfunction of T cells in HIV-1-infected individuals.

## Concluding remark

DNMTs are the enzymes which catalyze the CpG DNA methylation and have been reported to be over-expressed in various cancers. The mechanisms of DNMT over-expression are worthy of investigation. The transcriptional up-regulation on *DNMT* gene expression can be induced by Ras-c-Jun signaling pathway, Sp1 and Sp3 zinc finger proteins, wilms' tumour 1, homeobox B3 and various human viruses. Loss of transcriptional repression control on *DNMT* genes has also been reported. For example, p53 transcriptionally suppresses *DNMTs* through binding with Sp1 protein to the *DNMT* promoters. RB transcriptionally suppresses *DNMT1/3A* through binding with E2F1 protein to the *DNMT1* and *3A* promoters. FOXO3a binds to the *FOXO3a* DNA element of the *DNMT3B* promoter to repress *DNMT3B* transcription. In addition, overexpressed MDM2 may induce *DNMT1, DNMT3A,* and *DNMT3B* expression by negative control over p53, RB and FOXO3a. Low expressions of some miRs such as miR-29s, miR-143, miR-148a and miR-152 are associated with DNMT overexpression in various cancers. Several important post-translational modification including acetylation and phosphorylation have been reported to affect protein stability and activity of the DNMTs especially DNMT1. Therefore, drugs targeting DNMT protein inactivation and depletion, such as MDM2, AKT and CDKs inhibitors may prove to be a good therapeutic strategy for cancer treatment. Combined treatment with the known DNMT inhibitors such as decitabine could be a potential therapeutic strategy through epigenetic modulation warranting further investigation in cancer treatment.

## Abbreviations

DNMT: DNA methyltransferase; FOXO3a: forkhead O transcription factor 3a; HBx: hepatitis B virus X protein; HAUSP: herpesvirus-associated ubiquitin specific protease; hnRNP-U: heterogeneous nuclear ribonucleoprotein U; LMP1: latent membrane protein 1; miR: microRNA; NNK: nitrosamine 4-(methylnitrosamino)-1-(3-pyridyl)-1-butanone; RB: retinoblastoma; RGS: regulator of G protein signaling; TSG: tumor suppressor gene; UHRF1: ubiquitin-like containing PHD and RING finger domains 1.

## Competing interests

The authors declare that they have no competing interests.

## Authors’ contributions

RKL and YCW wrote the review. Both authors read and approved the final manuscript.
